# The role of single nucleotide polymorphisms related to iron homeostasis in mesothelioma susceptibility after asbestos exposure: a genetic study on autoptic samples

**DOI:** 10.3389/fpubh.2023.1236558

**Published:** 2023-10-24

**Authors:** Pierangela Grignani, Silvia Damiana Visonà, Maria Vittoria Fronda, Paola Borrelli, Maria Cristina Monti, Barbara Bertoglio, Adelaide Conti, Paolo Fattorini, Carlo Previderè

**Affiliations:** ^1^Department of Public Health, Experimental and Forensic Medicine, University of Pavia, Pavia, Italy; ^2^Laboratory of Biostatistics, Department of Medical, Oral and Biotechnological Sciences, G. d’Annunzio, University of Chieti, Chieti, Italy; ^3^Department of Medical and Surgical Specialities, Radiological Sciences and Public Health, Forensic Medicine Unit, ASST Spedali Civili of Brescia, Brescia, Italy; ^4^Department of Medicine, Surgery and Health, University of Trieste, Ospedale di Cattinara, Trieste, Italy

**Keywords:** asbestos, malignant mesothelioma, SNP, single base extension (SBE), iron metabolism

## Abstract

Asbestos-related diseases still represent a major public health problem all over the world. Among them, malignant mesothelioma (MM) is a poor-prognosis cancer, arising from the serosal lining of the pleura, pericardium and peritoneum, triggered by asbestos exposure. Literature data suggest the key role of iron metabolism in the coating process leading to the formation of asbestos bodies, considered to be both protective and harmful. Two sample sets of individuals were taken into consideration, both residing in Broni or neighboring cities (Northwestern Italy) where an asbestos cement factory was active between 1932 and 1993. The present study aims to compare the frequency of six SNPs involved in iron trafficking, previously found to be related to protection/predisposition to MM after asbestos exposure, between 48 male subjects with documented asbestos exposure who died of MM and 48 male subjects who were exposed to asbestos but did not develop MM or other neoplastic respiratory diseases (Non-Mesothelioma Asbestos Exposed – NMAE). The same analysis was performed on 76 healthy male controls. The allelic and genotypic frequencies of a sub-group of 107 healthy Italian individuals contained in the 1000 genomes database were considered for comparison. PCR-multiplex amplification followed by SNaPshot mini-sequencing reaction was used. The findings presented in this study show that the allelic and genotypic frequencies for six SNP markers involved in iron metabolism/homeostasis and the modulation of tumor microenvironment are not significantly different between the two sample sets of MM and NMAE. Therefore, the SNPs here considered do not seem to be useful markers for individual susceptibility to mesothelioma. This finding is not in agreement with previous literature.

## 1. Introduction

Asbestos-related diseases still represent a major public health problem all over the world. Among them, malignant mesothelioma (MM) is a highly aggressive, poor-prognosis cancer, arising from the serosal lining of pleura, pericardium and peritoneum, triggered by asbestos exposure.

Globally, 107,000 deaths from asbestos-related diseases per year have been estimated by the World Health Organization (WHO) (1). According to the rates per gender, mesothelioma is more common in men than in women (over 75% of the MM patients are male) probably due to a greater likelihood of occupational exposure to asbestos of the former (2). Even though the use of asbestos has been banned in 55 countries worldwide, the production and use of asbestos, still widespread all over the world, represents nowadays a major public health concern. In many countries the mining and use of asbestos is still allowed (the main consumers being Russia, China and Kazakhstan (1)). Typically, MM occurs thirty to fifty years after the first exposure to asbestos (3), therefore, even in countries in which asbestos is now banned, people are still suffering the consequences of asbestos exposure dating back to decades before. The reason for such a long latency is still unclear, although it has been suggested that it might be related to the time required for asbestos fibers to migrate from the lung to the pleural/peritoneal tissue (4).

Asbestos-related diseases are often brought to the forensic pathologist’s attention because of the high mortality rate of the MM and lung cancer, but also because of the complex legal implications related to possible responsibility of the manufacturers. In the legal context, the issue of a possible genetic predisposition to develop MM after asbestos exposure is of crucial importance, since a different individual susceptibility may change the strength of causal correlation between asbestos exposure and death due to MM and, consequently, the attribution of criminal responsibility.

Although MM has been considered for many years the paradigm of environmentally determined cancers, much evidence, reported in the literature, suggests a potential role of a genetic component in the etiology of this disease. This hypothesis is supported by a number of findings: (a) only a minority of asbestos-exposed subjects develop MM (5–17% of heavily exposed individuals) (5); (b) some subjects develop malignant mesothelioma following very low doses of asbestos exposure, whereas others, exposed to higher quantities, do not suffer from this disease (6); (c) there are frequent reports of MM familial clustering (7–11).

Many attempts have been made in order to identify the genetic substrate of mesothelioma predisposition. Studies carried out on families with a high incidence of mesothelioma found that a germline mutation in BRCA1-associated protein 1 (BAP1) and a following somatic mutation in the same locus originate the biallelic inactivation of the gene (10). BAP1 has several cell-intrinsic tumor suppressive functions, such as regulation of cell cycle and replication, gene transcription, DNA damage response, as well as a modulation in the inflammatory response to crocidolite (12). The role of BAP1 mutations in mesothelioma susceptibility has been subsequently confirmed by several other reports (13–15). Up to now, BAP1 is the only gene whose role in determining predisposition to mesothelioma is known (14).

Two genome-wide association studies (GWASs) on mesothelioma were carried out (16). Cadby et al. identified an association between a number of Single Nucleotide Polymorphisms (SNPs) in CRTAM, SDK1 and RASGRF2 genes, involved in cell migration and adhesion, and an increased risk to develop MM, but this result was not confirmed in further series of cases (16). Matullo et al. did not find any marker which reached the genome-wide significance threshold, but some interesting associations were pointed out: the main genes involved being SLC7A14, PVT1, MMP14 (17). Interestingly, the two studies did not produce homogeneous results.

Previous studies had reported an association between SNPs in GSTM1, XRCC3, SOD2 and EPHX and MM (5). The N-acetyltransferase 2 (NAT2) and manganese superoxide dismutase (MnSOD), involved in the organism’s defense against reactive oxygen species (ROS), have been reported to be associated with the risk of developing MM after asbestos exposure (18, 19). Other authors highlighted a correlation between SNPs in some genes involved in DNA repair systems and MM, such as XRCC3, a protein involved in repairing DNA breaks through homologous recombination, and ERCC1, a protein involved in nucleotide excision repair (20, 21). Senk et al. found that SNPs in aquaporin1 gene contribute to the risk of developing MM and may also influence the outcome of chemotherapy with cisplatin (22), while Strbac et al. pointed out an association between genetic variance in genes coding for matrix metalloproteinases and MM risk (23). Finally, Crovella et al. and Celsi et al. found a significant association between Fe-metabolism gene variants and protection against MM (24, 25). From our point of view, these last papers are of particular interest because the presence of iron on asbestos fibers (but, more importantly, their tendency to bind it in a biological environment) has been established to be crucial in determining the cytotoxicity and carcinogenic effect of asbestos fibers (26). Although its exact origin is still controversial and not fully understood, iron undoubtedly has a central role in the first tool of “defense” of the human organism against asbestos: the formation of asbestos bodies (AB). On the other hand, AB are known to exert a pro-inflammatory effect (27). AB consist of an inhaled fiber coated with iron and organic matter (mainly proteins, mostly ascribable to ferritin) (28). What is known about the mechanism of formation can be summarized as follows: when an asbestos fiber is introduced into the respiratory tract, macrophages try to phagocytise it. If a fiber is longer than 20 μm, a single cell is not able to ingest it entirely, and consequently the “frustrated phagocytosis” triggers a series of inflammatory mechanisms that somehow cause the accumulation of iron in the cells. Iron micelles appear in macrophages’ cytoplasm in proximity to the fiber, where the coating is formed by accumulation of these ferruginous micelles, together with homogeneous matrix material (28). Moreover, asbestos fibers have the intrinsic capacity to complex iron from the surrounding environment (29, 30). Indeed, a vicious cycle is established: the more iron the fiber attracts from the tissue, the more inflammation is triggered and, consequently, more iron is accumulated around the fiber.

Then, the Fe3+ on the fiber surface is reduced to Fe2+ by reductants (such as superoxide) in order to be internalized in the cells by a divalent metallic ion transporter (DMT). Reduced iron has the ability to produce oxidative stress. Once internalized into cells, iron must be safely accumulated. To be stored in ferritin (the principal and safer way of storage) iron must be oxidized again to Fe3+: this reaction happens directly inside the ferritin, which is able to catalyze it. Another way of iron storage consists of hemosiderin, a product of partial degradation of ferritin with a higher iron-to-protein ratio. Indeed, the asbestos bodies are composed mainly of ferritin and hemosiderin (28), with a very low presence of hematite and metallic iron, found to be well below 5% (31).

It is, therefore, evident that asbestos bodies have two faces: on the one hand, they can be regarded as an attempt of protection against asbestos, separating the cytotoxic fibers from the biological tissues; on the other hand, the formation of asbestos bodies around fibers implies an intrinsic cytotoxic and pro-inflammatory effect, given the generation of reactive oxygen species (ROS) (27, 30).

On the basis of the essential role of iron in the asbestos-induced cytotoxicity and cancerogenesis, and considering our SEM-EDS (Scanning Electron Microscope - Energy Dispersive X-ray Spectroscopy) observations, showing a marked variability in the number of AB in comparison to fibers (32), we reasoned that the different individual predisposition to develop mesothelioma might lie in genetic factors influencing the Fe-mechanisms mentioned above.

On the basis of the above cited literature, as well as the well-known role of iron-induced oxidative stress consequent to asbestos exposure, we decided to investigate the frequencies of six SNPs involved in iron metabolism/homeostasis and the modulation of tumor microenvironment in male MM patients and in male individuals exposed to asbestos but who died from other causes.

## 2. Materials and methods

### 2.1. Ethics statement

The project study has been approved by the reference Ethical Committee for the University of Pavia (Italy) (prot. N. 20190052849). All samples were immediately anonymized.

### 2.2. Samples

The samples used in this study were statistically dimensioned assuming a statistical power of at least 80%, identifying a minimum sample size of 43 subjects per group. The samples have been anonymized and divided into three distinct groups: (1) autoptic cases of subjects who died from pleural malignant mesothelioma (MM) with documented asbestos exposure (Mesothelioma Asbestos Exposed – MAE): Formalin-Fixed and Paraffin Embedded (FFPE) healthy heart tissue samples were selected from 48 male subjects who died because of MM. (2) Autoptic cases of subjects with documented asbestos exposure (Non-Mesothelioma Asbestos Exposed – NMAE): samples of FFPE healthy heart tissue were selected from 48 male subjects with a history of exposure to asbestos but who did not develop MM or other neoplastic diseases. 33 out of 48 subjects of this group had a diagnosis of asbestosis (confirmed histologically postmortem), according to the guidelines (33). For the other 15 subjects the history of exposure to asbestos was based on an accurate residential history. Subjects of groups 1 and 2, whose autopsies were performed between 2005 and 2018, used to live in the city of Broni (North-West Italy) or adjacent areas where an intense air dispersion of asbestos was present, according to epidemiological data (34, 35). These subjects lived in the same place and/or worked at the asbestos-cement plant located there, where asbestos-cement products were manufactured using a mixture of chrysotile and crocidolite (with small amount of amosite) between 1932 and 1993. Heart FFPE tissue was selected for the reason that cardiac muscle is a good DNA source useful to determine the germline status of the subjects exposed to asbestos, according to the selected markers, thus avoiding genetic alterations originating from the contamination from cancerous cells. All the autoptic samples were retrieved from the archive of the Legal Medicine and Forensic Sciences Unit of the University of Pavia. (3) A selection of DNA extracts from subjects without any known asbestos exposure (Healthy Controls – HC). Seventy-six samples of DNA extracted from the blood of healthy male subjects were selected from the case study analyzed at the Forensic Genetics laboratory at the Legal Medicine Unit of the University of Pavia. All living subjects gave their informed consent to use their biological sample for research purposes.

### 2.3. DNA extraction

The DNA extraction procedure applied to the FFPE samples is based on the use of a non-ionic and non-denaturing detergent, the Nonidet P40 (NP40; Nonylphenyl-polyethilene glycol). For each sample, 2–3 10 μm slices of healthy FFPE heart tissue were cut at the microtome; 300–500 μL of the non-ionic buffer described by Higuchi (36) were then added. The protocol for FFPE DNA extraction described by van Eijk et al. (37) was then followed: 20 μL of Proteinase K (10 mg/mL) were added and the samples incubated at 58°C for 4–5 h. The proteinase was then inactivated by boiling the samples at 100°C for 10 min; the samples were then centrifuged at 12,000 rpm for 10 min and transferred into a new Eppendorf tube. A negative control sample was included for every extraction to ensure the absence of contamination.

For samples of the control group, DNA extraction had been previously performed using the QIAmp® DNA Mini kit (QIAGEN), following manufacturer’s instructions or the phenol/chloroform DNA purification method. Negative control samples had always been included.

### 2.4. DNA quantification

2 μL of each FFPE DNA extract were quantified by Real Time PCR on a 7500 Real Time PCR System (Applied Biosystems, ThermoFisher Scientific) using the Quantifiler™ Duo DNA Quantification kit (Applied Biosystems, ThermoFisher Scientific) following manufacturer’s instructions. Instrument calibration was performed in duplicate, using a DNA standard at different concentrations (from 23 pg/μL to 50 ng/μL). Negative control (no template) samples were always included. The samples were analyzed with the HID Real Time PCR Analysis Software v. 1.1 (Applied Biosystems, ThermoFisher Scientific).

Samples of the healthy control (HC) group were quantified by the Quantus™ Fluorometer (Promega) and the QuantiFluor® ONE dsDNA System (Promega), following manufacturer’s instructions. The instrument calibration curve was calculated by quantifying standard samples as per manufacturer’s instructions.

### 2.5. Selected markers

Five SNPs markers from a group of 86 SNPs involved in iron metabolism/homeostasis were selected from the paper by Crovella et al. (24); three of them (rs76059597, rs2715631, and rs3747359) were found by the research group to be significantly associated with the risk of developing mesothelioma in subjects exposed to asbestos and were thus selected for the current study.

The polymorphism rs76059597 (T > C) is an intronic polymorphism located on the Ferritin heavy chain 1 (FTH1) gene, on Chromosome 11. This gene encodes the heavy subunit of ferritin, the major protein responsible for the intracellular iron storage. It is implicated in the oxidation of the iron ferrous form (Fe2+) to a less toxic form (Fe3+), thus allowing its safe storage within the protein (38).

The polymorphism rs2715631 (T > G) is located in an intronic region of the Transferrin (TF) gene on Chromosome 3. This gene encodes a glycoprotein involved in the transport of two iron ions, in the ferric form (Fe3+), through the bloodstream. Cellular uptake occurs by a receptor-mediated endocytosis, making iron available to be stored in ferritin to be used in metabolism or exported in the extracellular space (38).

The polymorphism rs3747359 (G > C) is a missense variant localized on the Hephaestin (HEPH) gene, on Chromosome X. The HEPH gene encodes a protein of the multicopper oxidase family, with a ferroxidase activity for oxidizing the ferrous iron in the ferric form. It is involved in the cellular iron ion homeostasis, and in particular in the iron transport from the cellular compartment (39). The SNP results in a non-synonymous aminoacidic substitution (Asp/His) at position 568 of the Hephaestin protein (40).

In addition, two other SNPs from the same paper, rs224575 (T > C) and rs224589 (T > G), were selected among the genes involved in iron homeostasis which were found to be overexpressed in mouse mesothelioma tissues (41). These are localized in an intronic region of the solute carrier family 11 member 2 (SLC11A2) gene, on Chromosome 12. The product of the gene is a proton-coupled divalent metal ion transporter, DMT-1, which is involved in iron absorption.

Finally, a sixth SNP was chosen: rs243865 (C > T), a polymorphism in the promoter sequence, known to be associated with the proliferation and evolution of malignant mesothelioma (42). The SNP is located on the matrix metallopeptidase 2 (MMP2) gene, on Chromosome 16. Matrix metalloproteinases (MMPs) are a family of zinc-containing endopeptidases involved in the cleavage of the components of the extracellular matrix and basement membrane (43). Studies in the literature identified MMPs as having a role in modulating tumor microenvironment and, specifically MMP-2 and MMP-9, in tumor angiogenesis, invasion, and metastasis (43, 44). Recent literature data suggested MMP-2 rs243865T to have a protective role in malignant pleural mesothelioma (22).

### 2.6. DNA amplification

DNA amplification of the selected markers was performed by PCR multiplex; no standardized kit was available for the selected SNPs. Primers were designed using the software Primer3 (45–47) and then tested for specificity on BLAST (48) and for hairpins and dimers on AutoDimer (49). To verify the genotypes of the selected SNPs, each marker was initially amplified in a singleplex assay, using 0.5 ng of the single source human genomic DNA 2800 M (Promega). Then, all regions containing the SNPs of interest were simultaneously amplified in a PCR multiplex reaction which was set up in a final volume of 25 μL containing 5 μL of 5X GoTaq Flexi buffer (Promega), 1.5 mM of MgCl_2_, 200 μM of each dNTP, 1 U GoTaq Hot Start Polymerase (Promega), and 2.5 μL of premixed primers (10X). Primer sequences and final concentrations in the master mix are shown in Supplementary Table S1.

To each sample, 0.5–1 ng of DNA were added. The single source human genomic DNA 2800 M (Promega) and a negative control sample (no template DNA) were always included in each multiplex PCR amplification. PCR thermal cycling conditions were the following: pre-incubation step at 95°C for 2 min, then 30 cycles of denaturation at 94°C for 30 s, annealing at 56°C for 30 s, extension at 72°C for 1 min and finally an elongation step at 72°C for 5 min. To check the correct amplification and the corresponding size of the PCR products, all samples were separated by electrophoresis on a 2% agarose gel in TAE buffer (0.5X).

### 2.7. Single base extension assay (SBE)

To eliminate residual unincorporated primers from the PCR multiplex reaction, 1 μL of Exo I and 1 μL of SAP (Applied Biosystem, ThermoFisher Scientific) were added to 2–4 μL of amplified DNA for each sample on the basis of the evaluation of band intensity after gel electrophoresis. The samples were then treated at 37°C for 10 min, and finally at 80°C for 10 min.

The multiplex primer extension reactions were performed, using the SNaPshot™ Multiplex Kit (Applied Biosystem, ThermoFisher Scientific). The Single Base Extension reaction was performed in a final volume of 7 μL by adding 1.5–3 μL of ExoSAP purified DNA, 0.7 μL of 10X primer mix, and 2 μL of SNaPshot™ Multiplex Ready Reaction Mix. Specific extension primers used in the SBE were designed with their 3′ base corresponding to the base immediately before the SNP investigated in this study to accurately detect the corresponding genotypes. Poly(T) tails were added to spatially separate the SNPs during capillary electrophoresis. Sequences and final concentrations of the extension primers used in this reaction are reported in Supplementary Table S2.

The single source human genomic DNA 2800 M (Promega) and a negative control sample (no template DNA) were always included in each primer extension reaction. The SNaPshot reactions were then performed for 25 cycles at 96°C for 10 s (denaturation phase), 50°C for 5 s (annealing phase), and 60°C for 30 s (extension phase).

After the SBE reaction, the samples were again purified by adding 1 μL of SAP (Applied Biosystem, ThermoFisher Scientific) to 2–4 μL of primer extended products in order to remove unincorporated ddNTPs. The samples were treated at 37°C for 60 min and at 80°C for 15 min to inactivate the enzyme.

For each sample, 10 μL of formamide and 0.1 μL of GeneScan™ 120 LIZ® Size Standard (Applied Biosystem, ThermoFisher Scientific) were added to 1–3 μL of purified primer extended products. After denaturation, the samples were separated on an ABI PRISM 310 Genetic Analyzer automatic sequencer, and the resulting data were analyzed with the GeneMapperID® ver 3.2.1 software (Applied Biosystem, ThermoFisher Scientific).

### 2.8. Statistical analysis

Statistical analysis was performed using the open-source software PLINK (v1.07) (50) and Stata 15 (51). Hardy–Weinberg Equilibrium was tested for all three genotyped groups (significant value *p* < 0.001). To compare, pairwise, allele and genotype frequencies between the three genotyped groups, and also between the groups and the Tuscan Italian population in 1000 Genomes (52), the Chi-Squared and Fisher Exact tests were employed as appropriate and *p*-value <0.05 was considered statistically significant. Odds ratios (OR) and 95% confidence intervals (95% CI) were also calculated for statistically significant results.

## 3. Results

### 3.1. Subjects’ characteristics

In total, we included 48 male patients exposed to asbestos who died from malignant mesothelioma (MM), 48 male subjects with ascertained asbestos exposure who died from other non-neoplastic causes (NMAE), and 76 healthy males (HC) as a control group. All the subjects were selected among males (MM is more frequent among males), thus avoiding a potential confounding factor in this phase of the study.

Demographic and clinical characteristics of MM and NMAE subjects are summarized in Table 1.

**Table 1:** Anamnestic and demographic data of MM and NMAE subjects.

	MM (*n* = 48)	NMAE (*n* = 48)
Age at death (mean)	69.6 (12.35)	77.5 (11.4)
Age at death (range)	40–94	40–95
Cause of death		
Epithelioid MM	33	0
Sarcomatoid MM	3	0
Biphasic MM	12	0
Traumatic death	0	11
Natural death	0	37
Asbestos exposure		
Occupational*	34	36
Anthropogenic environmental**	14	12
Cigarette smoking		
Yes	25	26
No	11	7
Unknown	12	15
Median survival since diagnosis (months)	12	NA
Median latency since first exposure (years)	46	NA
Age of histological samples	10	13

^*^ Refers to people who worked in contact with asbestos even if, in some cases, not on daily basis. ^**^ Refers to people who lived in an area with air dispersed asbestos from the asbestos-cement plant.

### 3.2. DNA quantification

#### 3.2.1. FFPE Molecular DNA quantification (qPCR)

DNA was recovered from the FFPE heart tissues belonging to the two sets of asbestos exposed subjects (MM and NMAE), according to the NP40 DNA extraction protocol as described in paragraph 2.3. Each sample was then quantified using the Quantifiler™ Duo DNA Quantification kit (Applied Biosystems, ThermoFisher Scientific), which provided human DNA amounts according to the autosomal and Y-chromosome probes in a range from 23 pg/μL to 50 ng/μL. The molecular quantifications yielded lower DNA amounts for the autosomal probe, compared to the Y-specific one, likely due to the inverse relations between DNA degradation pattern of the FFPE samples and the size (in bp) of the qPCR products. For this reason, since all samples were of male gender, we decided to normalize the DNA amounts for the following PCR amplifications according to the shorter Y-specific probe, more efficient in the amplification of degraded DNA. In Table 2 the mean, median, and min/max values of the amplifiable DNA amounts are reported for the two sets of FFPE heart tissue samples. A great variability in the DNA amounts recovered from the FFPE samples was reported, as confirmed by the min and max values described in Table 2. A correlation between the DNA amounts and the age of both sets of FFPE samples, in terms of time elapsed from the year of tissue inclusion (from 2005 to 2018) and the present analysis, was investigated but no significant statistical values were found. Inter-individual anatomic differences between the sampled tissues or in postmortem intervals (PMI) or in formalin fixation times could explain the variations in the DNA amounts. Most of the FFPE samples showed DNA amounts above the lowest point of the calibration curve (23 pg/μL) (see Table 2). The absence of PCR inhibition in the FFPE samples was assessed by the correct amplification of the internal positive control (IPC).

**Table 2:** Molecular DNA quantification (pg/μL) results obtained, according to the Y-probe of the Quantifiler™ Duo DNA Quantification kit (Applied Biosystems, ThermoFisher Scientific), for the set of subjects exposed to asbestos analyzed in the present study.

	Malignant Mesothelioma (MM) *N* = 48 individuals	Non-Mesothelioma Asbestos Exposed (NMAE) *N* = 48 individuals
*Mean*	468	288
*Median*	341	100
*Range (Min-Max)*	13–2308	12–1762
*N. samples > LOQ*	47	43

For each set of FFPE samples, the mean, median, and the range (min-max) DNA amounts are reported. The last row shows the number of FFPE samples which yielded DNA amounts above the lowest point of the qPCR calibration curve (Limit of Quantification, LOQ, 23 pg/μL).

#### 3.2.2. Control samples: fluorimetric DNA quantification

A fluorometric quantification of the 76 healthy DNA control samples provided the following results: mean, median, range (min-max) DNA amounts were 91, 43, 3–338 ng/μL, respectively.

### 3.3. Genetic analysis

#### 3.3.1. Multiplex PCR amplification and SBE assay

**Figure 1:**
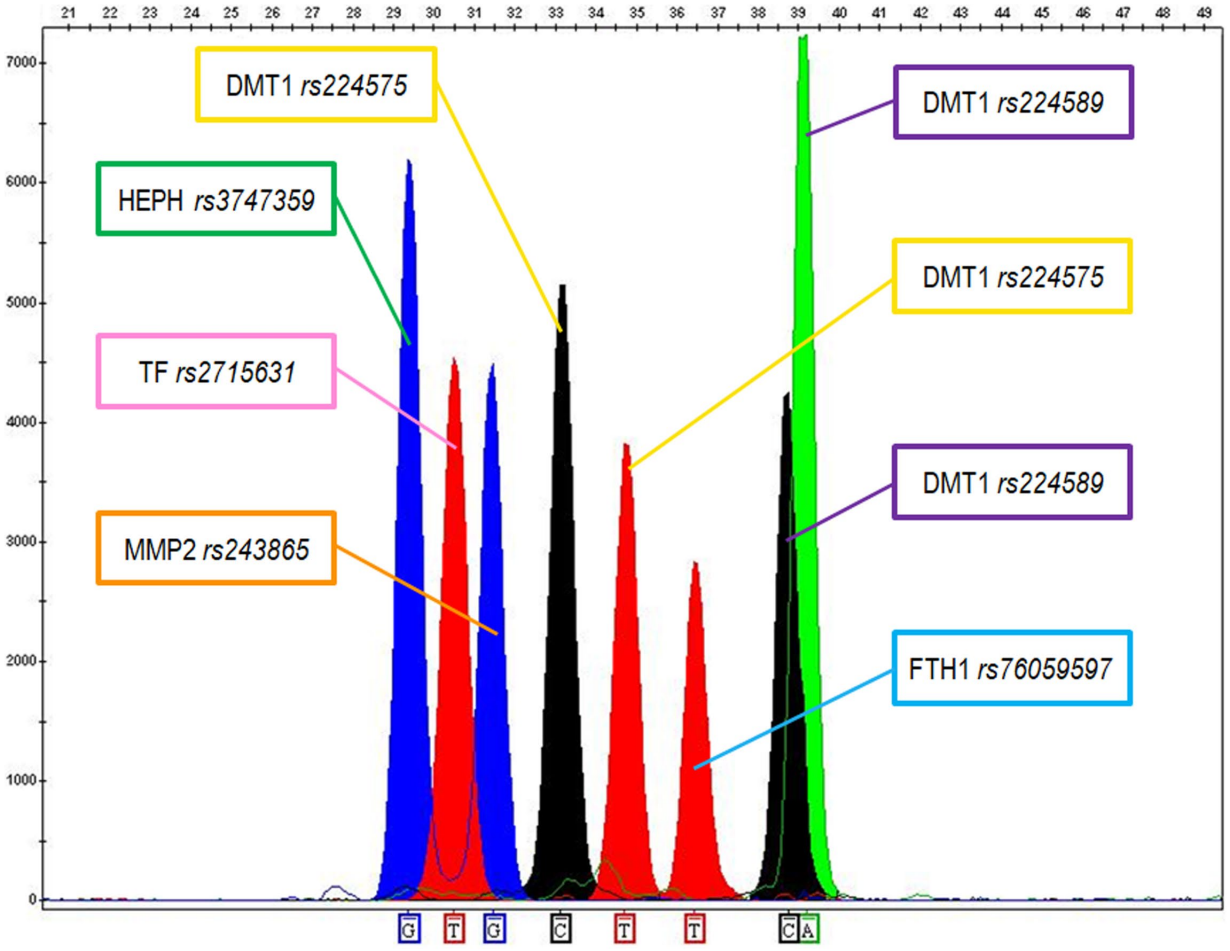
Electropherogram showing the genetic typing of the 2800 M control DNA according to the 6 SNPs. The following genotypes were recorded: G for Haephaestin (HEPH rs3747359); TT for Transferrin (TF rs2715631); GG for MMP2 (MMP2 rs243865); CT for DMT1 (DMT1 rs224575); TT for Ferritin (FTH1 rs76059597); CA for DMT1 (DMT1 rs224589).

FFPE tissues represent a valid source of specimens for histological diagnosis and for the assessment of included tissues’ genetic features. However, it is well known that the commonly used fixative formaldehyde leads to the generation of DNA/RNA-proteins linkages, which can block polymerase during PCR, or to the degradation of the nucleic acids (53). To overcome this problem, we picked primer pairs able to originate small-sized amplicons in the range of 100–120 bp for the selected SNP markers, consistent with the molecular weight of a severely degraded/modified DNA. Each SNP was initially amplified in a single PCR reaction to check the incorporation of the expected nucleobases and the corresponding electrophoretic mobility; then a multiple PCR assay was set up. Specific probes were used to target the six selected SNPs involved in iron metabolism/homeostasis and the modulation of tumor microenvironment in a SBE assay. The extended probes, which incorporated the corresponding nucleotide bases, were separated through capillary electrophoresis producing a separation of the allelic peaks as the one shown in Figure 1 for the DNA control sample 2800 M.

**Figure 2:**
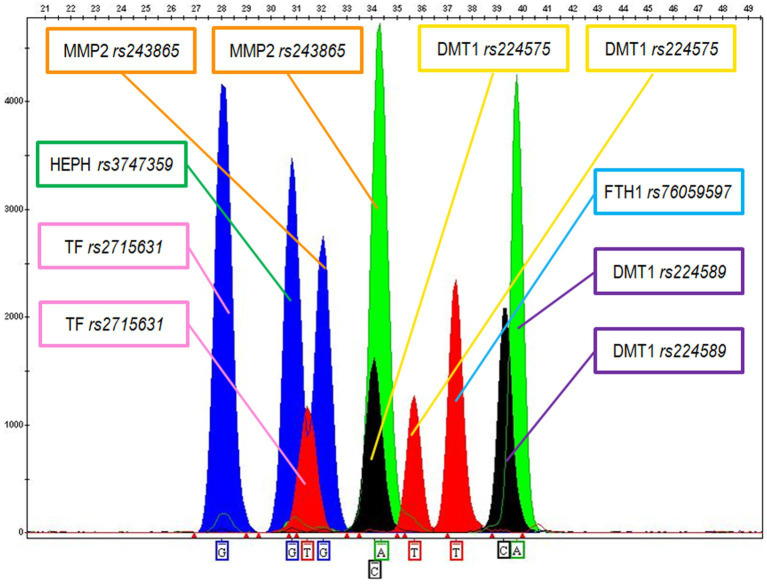
Electropherogram showing the following genotypes for MM sample X: G for Haephaestin (HEPH rs3747359); GT for Transferrin (TF rs2715631); GA for MMP2 (MMP2 rs243865); CT for DMT1 (DMT1 rs224575); TT for Ferritin (FTH1 rs76059597); CA for DMT1 (DMT1 rs224589).

The optimal DNA amount for FFPE sample amplifications was estimated between 0.5 and 1 ng. Most of the FFPE tested samples produced good quality SBE profiles with clearly detectable peaks (see Figure 2), while a limited number of samples showed lower quality profiles with a significant reduction of the allele peak heights for some of the genetic markers and for this reason were re-extracted and/or re-amplified using an increased amount of DNA.

#### 3.3.2. Allelic and genotypic frequencies and genetic association

In Table 3 the allelic and genotypic frequencies of the six SNPs analyzed are reported. In the same table, the allelic and genotypic frequencies of a subgroup of 107 healthy Italian individuals contained in the 1000 genomes database (54) are shown for comparison.

**Table 3:** Allelic and genotypic frequencies (genetic typing) of the SNPs markers in the selected population samples: MM- subjects who died of malignant mesothelioma; NMAE – subjects with documented asbestos exposure who died for causes different from mesothelioma; HC – healthy controls subjects without documented asbestos exposure; 1000 genomes Italians (TSI) – healthy subjects from Tuscany (Italy) whose genetic data are in the 1000 genomes database.

Markers	Genetic typing		MM *N* = 48	NMAE *N* = 48	HC *N* = 76	1000 genomes Italians (TSI) *N* = 107
**FTH1 rs76059597**	Alleles	T	92 (95.83%)	94 (97.92%)	146 (96.05%)	207 (96.73%)
		C	4 (4.17%)	2 (2.08%)	6 (3.95%)	7 (3.27%)
	Genotypes	TT	44 (91.67%)	46 (95.83%)	70 (92.11%)	100 (93.46%)
		TC	4 (8.33%)	2 (4.17%)	6 (7.89%)	7 (6.54%)
		CC	0 (0%)	0 (0%)	0 (0%)	0 (0%)
**TF rs2715631**	Alleles	T	65 (67.71%)	68 (70.83%)	110 (72.37%)	165 (77.10%)
		G	31 (32.29%)	28 (29.17%)	42 (27.63%)	49 (22.90%)
	Genotypes	TT	26 (54.17%)	22 (45.83%)	41 (53.95%)	66 (61.68%)
		TG	13 (27.08%)	24 (50.00%)	28 (36.84%)	33 (30.84%)
		GG	9 (18.75%)	2 (4.17%)	7 (9.21%)	8 (7.48%)
**MMP2 rs243865**	Alleles	G	71 (73.96%)	69 (71.88%)	121 (79.61)	158 (73.83%)
		A	25 (26.04%)	27 (28.12%)	31 (20.39%)	56 (26.17%)
	Genotypes	GG	27 (56.25%)	24 (50.00%)	47 (61.84%)	58 (54.21%)
		GA	17 (35.42%)	21 (43.75%)	27 (35.53%)	42 (39.25%)
		AA	4 (8.33%)	3 (6.25%)	2 (2.63%)	7 (6.54%)
**DMT1 rs224575**	Alleles	T	37 (38.54%)	36 (37.50%)	67 (44.08%)	89 (41.59%)
		C	59 (61.46%)	60 (62.50%)	85 (55.92%)	125 (58.41%)
	Genotypes	TT	6 (12.50%)	7 (14.58%)	16 (21.05%)	19 (17.76%)
		CT	25 (52.08%)	22 (45.84%)	35 (46.06%)	51 (47.66%)
		CC	17 (35.42%)	19 (39.58%)	25 (32.89%)	37 (34.58%)
**DMT1 rs224589**	Alleles	A	21 (21.88%)	22 (22.92%)	45 (29.61%)	62 (28.97%)
		C	75 (78.12%)	74 (77.08%)	107 (70.39%)	152 (71.03%)
	Genotypes	AA	0 (0%)	2 (4.17%)	6 (7.89%)	7 (6.54%)
		CA	21 (43.75%)	18 (37.50%)	33 (43.42%)	48 (44.86%)
		CC	27 (56.25%)	28 (58.33%)	37 (48.69%)	52 (48.60%)
**HEPH rs3747359**	Alleles	G	48 (100%)	48 (100%)	76 (100%)	161 (100%)
		C	0 (0%)	0 (0%)	0 (0%)	0 (0%)
	Genotypes	GG Female	-	-	-	54 (50.47%)
		G Male	48 (100%)	48 (100%)	76 (100%)	53 (49.53%)

In brackets the relative percentages. N: number of individuals.

The genotypic frequencies of the three sample sets of subjects analyzed in this study were preliminary tested using the software PLINK (ver. 1.07) (50) and proved to be in Hardy–Weinberg equilibrium, being thus suitable for following statistical comparisons.

The SNP rs3747359 in the Hephaestin (HEPH) gene on Chromosome X was excluded from the statistical comparisons, as it was found to be non-polymorphic in our three sample sets and in the subgroup of healthy Italians contained in the 1000 genomes database (see Table 3).

Allelic and genotypic frequencies in the group of 48 subjects who developed mesothelioma (MM) and in the group of 48 individuals exposed to asbestos but who died for causes different from mesothelioma (NMAE) were compared.

The results of the statistical test, reported in Table 4, showed that no statistically significant difference in allelic and genotypic distribution was found for markers except for SNP rs2715631 in the Transferrin gene, according to the genotypic model (*p*-value = 0.018).

**Table 4:** Results of the Pearson’s Chi-Squared test (genotypic and allelic models) between the mesothelioma (MM) and exposed to asbestos (NMAE) groups.

Marker (SNP)	Model	Test	χ^2^	*p*-value
**FTH1** **(rs76059597)**	Genotypic	Fisher	–	0.677
	Allelic	Fisher	–	0.683
**TF** **(rs2715631)**	**Genotypic**	**χ2**	**8.06**	**0.018**
	Allelic	χ^2^	0.22	0.639
**MMP2** **(rs243865)**	Genotypic	Fisher	–	0.730
	Allelic	χ^2^	0.11	0.745
**DMT1** **(rs224575)**	Genotypic	χ^2^	0.38	0.827
	Allelic	χ^2^	0.02	0.882
**DMT1** **(rs224589)**	Genotypic	Fisher	–	0.442
	Allelic	χ^2^	0.03	0.863

In bold is reported the statistically significant model and the corresponding *p*-value.

The significance of this result was further investigated considering the dominant and recessive models, whose results are reported in Table 5. The recessive model showed a statistically significant *p*-value = 0.025 with an odd ratio (OR) = 5.31 and a 95% CI between 1 and 26, meaning that subjects with the recessive genotype GG are about 5 times more prone to develop mesothelioma compared to the individuals showing the other two genotypes (TT and TG). Notwithstanding, the confidence interval points out that, at the 95% level, the real risk value of developing mesothelioma in those individuals is very variable, being included between 1 and 26 times.

**Table 5:** Results of the Pearson’s Chi-Squared test (dominant and recessive models) for SNP rs2715631 between the mesothelioma (MM) and exposed to asbestos (NMAE) groups.

Marker (SNP)	Genetic model	Test	χ^2^	*p*-value	OR	95%CI
**TF** **(rs2715631)**	Dominant	χ2	0.666	0.41	–	–
	**Recessive**	**χ**^**2**^	**5.031**	**0.025**	**5.31**	**1–26**

In bold are reported the statistically significant model, the corresponding *p*-value, the odd ratio (OR) and the confidence interval (CI) at the 95% level.

No statistically significant differences for allelic and genotypic frequencies were observed between the mesothelioma (MM) and the healthy control (HC) groups and neither between MM and the subgroup of healthy Italians in the 1000 genomes database. Similarly, no differences were found between the NMAE and the healthy controls (HC) and between the NMAE and the Italian subjects in the 1000 genome database. Finally, allelic and genotypic frequencies were compared between the healthy controls (HC) and the 1000 genomes Italian subgroup, not supporting statistically significant differences.

## 4. Discussion

FFPE samples have always been traditionally regarded as not suitable for molecular and genetic analysis, as nucleic acids are inevitably damaged by formalin fixation. Nevertheless, recently the methods for extraction and analysis of DNA, RNA, and proteins have considerably improved, allowing molecular genetics studies using FFPE samples (55, 56). Even molecular diagnostic studies and retrospective GWA studies have been conducted using FFPE samples, providing valuable results (57). It is a trivial consideration that DNA extracted from FFPE samples produces lower quality profiles than those from fresh tissue (58). The main cause of alteration in the DNA structure is represented by the fixation in formaldehyde, the active component of the formalin, which cross-links the DNA and the surrounding proteins (59) and causes the breaks in the sequences (60, 61). Furthermore, both the pH and the duration of fixation with formalin affect the quality of the nucleic acid (62).

Besides formalin fixation, we must consider that we are dealing with autoptic samples, and therefore with the postmortem degradation of nucleic acids occurring before formalin fixation. Following cell death, endogenous nucleases initiate the DNA degradation (63). The rate at which DNA is degraded by endogenous nucleases depends on various factors, such as the tissue, the expression of enzymes, temperature, and pH (64).

Even non-enzymatic modifications of the DNA primary structure (N-glycosidic bonds breaking, oxidations, deaminations, and methylations) also occur (63).

Despite the above illustrated difficulties, FFPE samples often represent the only available source of information about human deceased patients. Therefore, retrospective, observational studies on archived samples are of essential importance, especially when we are dealing with rare tumors like MM.

Consistently with the results of the above cited studies, the extraction of DNA from FFPE samples provided sufficient quantities of DNA in all the subjects. In addition, the DNA extracted from our heart samples offered sufficient quality for SNPs analysis.

Similarly, the approach of molecular characterization of the six SNPs markers associated with iron metabolism and the modulation of tumor microenvironment, using the SNaPshot mini-sequencing method and subsequent separation in capillary electrophoresis, produced electropherograms of clear interpretation and, consequently, reliable genetic typing of the samples was obtained in the study.

Overall, in the present study we did not find any significant difference in allelic and genotypic frequencies of the above described SNPs between the group of 48 male subjects who developed mesothelioma (MM) and the group of 48 male individuals exposed to asbestos but who died from causes different from mesothelioma (NMAE).

Only the genotypic model of SNP rs2715631 in the Transferrin gene seemed to show a significant difference, suggesting an increased susceptibility to MM of the carriers of the genotype GG, compared to the other two, after asbestos exposure. Yet, considering the confidence interval, this difference cannot be confirmed due to the wide range of the possible odds ratio (from 1 to 26). This large interval can be originated by the limited number of subjects sampled in the two groups.

The results presented in this study are not in agreement with the data reported by Crovella et al. (24) on very similar sets of samples. The above authors analyzed FFPE archived autopsy heart tissue belonging to 77 individuals who died due to mesothelioma (MM) and 48 individuals exposed to asbestos but who did not develop MM or other neoplastic respiratory diseases (NMAE), and found three genotypes (C/C, G/G, C/C for ferritin, transferrin, and hephaestin, respectively) significantly associated with a protection against mesothelioma development. On the opposite hand, our data showed a substantial homogeneity in allelic and genotypic frequencies for all the six markers considered in this study both in population samples of individuals exposed to asbestos (MM and NMAE) and in healthy controls (HC and 1000 genomes), with a weak and uncertain risk value of developing mesothelioma for Transferrin genotype GG. One of the most striking differences was the one recorded for SNP rs3747359 in the Hephaestin (HEPH) gene; in our three sample sets, as well as in the subgroup of healthy Italians contained in the 1000 genomes database and even in the European populations described in (39), only allele G was identified, thus representing a non-polymorphic marker, while in the paper by Crovella et al. allele C reached the remarkable frequency of 37% in the set of individuals exposed to asbestos who did not develop mesothelioma. Most of the differences were, however, related to this last set of samples, whose allelic and genotypic frequencies did not follow the Hardy–Weinberg equilibrium, as stated by Crovella and coworkers themselves, and for this reason should not be compared to other data. More in detail, while the MM populations samples by Crovella and the MM series described in the present study showed very similar allelic and genotypic frequencies for the ferritin, transferrin, and hephaestin markers (*p* values >0.077), the NMAE series analyzed by Crovella clearly showed very different allelic and genotypic frequencies. This is a very difficult finding to explain as a different distribution of allelic and genotypic frequencies should be speculated in two numerically and quite homogeneous Italian population samples exposed to asbestos which were only 300 km away from each other. Another explanation could be found in the limited number of MM and NMAE subjects “enrolled” in the present study. In fact, this is a retrospective study based on archived autoptic samples sometimes hard to retrieve and from which it was sometimes difficult to get good quality DNA for the molecular analyses. In our study the number of FFPE tissues and the corresponding control samples was dimensioned to get a statistical power of at least 80%, which is considered a good value in detecting a difference as statistically significant.

Finally, it is worth reporting that different molecular typing methodologies were used to score the genotypes in the present paper and in the one by Crovella et al. (24). We systematically characterized all samples (exposed and controls) using the mini-sequencing technique (SBE), while Crovella analyzed the MM and the NMAE equivalent samples with a Veracode Chips and Bead Array technology on the iScan system (Illumina San Diego, CA), and the control samples with a TaqMan qPCR assay. Even degradation and modifications of the DNA primary structure could explain some of the differences discussed in the present paper; in fact, the quality of the genetic substrate extracted from the FFPE tissue might affect the outcome of the DNA typing methodology (63, 64).

Interestingly, the time elapsed since the fixation process did not appear to influence the DNA quality, contrary with previous literature (61). This result suggests that the damage induced by postmortem degradation and fixation is not significantly worsened by the subsequent storing time of the FFPE.

## 5. Conclusion

In conclusion, the findings presented in this study showed that the allelic and genotypic frequencies for six SNP markers involved in iron metabolism/homeostasis and the modulation of tumor microenvironment are substantially homogeneous in the two following sample sets of individuals who lived in Broni or neighboring cities (Northwestern Italy), where an asbestos cement factory was active from 1932 to 1993: (a) subjects who died of malignant mesothelioma (MM) with documented asbestos exposure; (b) subjects with a documented history of exposure to asbestos but who did not develop MM or other neoplastic respiratory diseases. Only a very weak predisposition to develop mesothelioma was found for the recessive genotype GG when SNP rs2715631 in the Transferrin gene data were statistically compared in these two groups. In addition, the same allelic and genotypic distribution found in MM were seen even in the healthy control samples and in the subset of Italians contained in the 1000 genomes database.

Therefore, not in agreement with previous literature (24), based on the series analyzed in the present report, the SNPs considered in this study do not seem to be useful markers for individual susceptibility to develop mesothelioma. On this basis, we have to conclude that this approach, even though very appealing given the central role of iron trafficking molecules in the response to asbestos, does not seem to be promising in addressing the complex problem of genetic susceptibility to MM.

## Data availability statement

All relevant materials of the present study is available upon request from the corresponding author to interested researchers.

## Ethics statement

The studies involving humans were approved by Comitato di Bioetica Policlinico San Matteo di Pavia. The studies were conducted in accordance with the local legislation and institutional requirements. The ethics committee/institutional review board waived the requirement of written informed consent for participation from the participants or the participants' legal guardians/next of kin because this study was conducted retrospectively on archive samples taken from deceased people.

## Author contributions

PG, SDV, and CP designed the study. PG and MVF collected and organized the data. PB and MCM performed the statistical analysis. CP and PF supervised the work. PG and BB designed tables and images. CP and SDV wrote the original draft. CP, PF, and AC reviewed and edited the draft. All authors contributed to the article and approved the submitted version.

## In memoriam

This work is dedicated to the memory of Antonio Osculati, who died on 11th November 2020. He participated and supervised the study and supported the team since the beginning of this project.
