# Supplementary material for: The role of single nucleotide polymorphisms related to iron homeostasis in mesothelioma susceptibility after asbestos exposure: a genetic study on autoptic samples
Source: Front Public Health. 2023 Oct 24;11:1236558. doi: 10.3389/fpubh.2023.1236558 (PMC10628177; doi:10.3389/fpubh.2023.1236558)
Supplement: Supplementary file 1 [file Table_1.docx]

Supplementary Material

The role of single nucleotide polymorphisms related to iron homeostasis in mesothelioma susceptibility after asbestos exposure: a genetic study on autoptic samples)

**Pierangela Grignani^*^, Silvia Damiana Visonà^*^, Maria Vittoria Fronda, Paola Borrelli, Maria Cristina Monti, Barbara Bertoglio, Adelaide Conti, Paolo Fattorini, Carlo Previderè**

*** Correspondence:**Silvia D. Visonà

silviadamiana.visona@unipv.it

# Supplementary table 1: Primer sequences, lengths of the amplicons (in bp) and final concentrations (FC) of the markers amplified in the multiplex PCR reaction

| **SNP** | **Direction** | **Primer sequence** | **Primer length (bp)** | **Fragment length (bp)** | **FC (µM)** |
| --- | --- | --- | --- | --- | --- |
| FTH1  rs76059597 | Forward | tgcacagaaaacttacagccag | 22 | 102 | 0.2 |
|  | Reverse | atacaggagcagggaggaga | 20 |  |  |
| TF  rs2715631 | Forward | gattttctctgctgagtgtgcc | 22 | 100 | 0.2 |
|  | Reverse | tccttgaagcagcctttcca | 20 |  |  |
| HEPH  rs3747359 | Forward | cacaaattctggcctggtgg | 20 | 103 | 0.6 |
|  | Reverse | gcaggctttccagccagata | 20 |  |  |
| MMP2  rs243865 | Forward | tccagtgcctcttgctgttt | 20 | 113 | 0.2 |
|  | Reverse | tgagctgagacctgaagagc | 20 |  |  |
| DMT1  rs224575 | Forward | aaccaatctacccacaattactga | 24 | 104 | 0.4 |
|  | Reverse | gccccgcttcaacaaaagac | 20 |  |  |
| DMT1  rs224589 | Forward | ccaacatgcagggtggagaa | 20 | 100 | 0.2 |
|  | Reverse | ctggagcagtggctggattt | 20 |  |  |

# Supplementary table 2: Sequences of the extension primers used to target the selected SNPs markers and the corresponding final concentrations in the SNaPshot reaction. The non-specific tail poly(T) is underlined.

| **SNP** | **Strand** | **Tail and extension primer sequences** | **Primer length (nucleotides)** | **Sequence variation** | **FC (µM)** |
| --- | --- | --- | --- | --- | --- |
| FTH1  rs76059597 | Forward | ttttttgagagggcgctggagtactgaccc | 30 | T/C | 0.1 |
| TF  rs2715631 | Forward | agtgtgcctggctgatcttt | 20 | T/G | 0.2 |
| HEPH  rs3747359 | Forward | tgcagggctggtgccttgggtgca | 24 | G/C | 0.6 |
| MMP2  rs243865 | Reverse | ttttgagacctgaagagctaaagaggt | 27 | G/A | 0.1 |
| DMT1 rs224575 | Forward | tttcgctccctgaagtcggttaggtta | 27 | T/C | 0.4 |
| DMT1 rs224589 | Reverse | ttttttttttcccctgtccttttaagcacataatac | 36 | A/C | 0.2 |
